# Using Multiple Barometers to Detect the Floor Location of Smart Phones with Built-in Barometric Sensors for Indoor Positioning

**DOI:** 10.3390/s150407857

**Published:** 2015-03-31

**Authors:** Hao Xia, Xiaogang Wang, Yanyou Qiao, Jun Jian, Yuanfei Chang

**Affiliations:** 1 The Institute of Remote Sensing and Digital Earth, No.20 Datun Road, Chaoyang District, Beijing 100101, China; E-Mails: qiaoyy@radi.ac.cn (Y.Q.); jianjun@radi.ac.cn (J.J.); changyf@radi.ac.cn (Y.C.); 2 Sichuan Geological Survey, No.25 Renmin Road, Chengdu 610081, China; E-Mail: rshunter@126.com

**Keywords:** floor positioning, barometer, smart phones

## Abstract

Following the popularity of smart phones and the development of mobile Internet, the demands for accurate indoor positioning have grown rapidly in recent years. Previous indoor positioning methods focused on plane locations on a floor and did not provide accurate floor positioning. In this paper, we propose a method that uses multiple barometers as references for the floor positioning of smart phones with built-in barometric sensors. Some related studies used barometric formula to investigate the altitude of mobile devices and compared the altitude with the height of the floors in a building to obtain the floor number. These studies assume that the accurate height of each floor is known, which is not always the case. They also did not consider the difference in the barometric-pressure pattern at different floors, which may lead to errors in the altitude computation. Our method does not require knowledge of the accurate heights of buildings and stories. It is robust and less sensitive to factors such as temperature and humidity and considers the difference in the barometric-pressure change trends at different floors. We performed a series of experiments to validate the effectiveness of this method. The results are encouraging.

## 1. Introduction

Following the popularity of smart phones and the development of mobile Internet, the demands for accurate indoor positioning have rapidly grown recently. In addition to traditional map and navigation applications, location information becomes necessary for some applications such as social networking and e-commerce. Because we spend most of our time working and living indoors, a high-precision indoor positioning system (IPS) is important in many applications.

Global Positioning System (GPS) has been used in various fields, but it cannot be used indoors [1]. In indoor environments, satellite signals cannot be used because they are highly attenuated by the walls of buildings. Furthermore, GPS signals that could be received propagate via a very complex propagation channel, *i.e.*, not through a line-of-sight path; thus, the propagation time cannot be directly transformed into distance. 

Some researchers have proposed IPS schemes based on the Earth’s magnetic field [2]. They used the magnetic field characteristics of buildings to position smart phones with magnetic sensors indoors. However, they focused on the positioning in one floor in a building and did not pay much attention to the positioning between floors. Majority of previous localization approaches employed Received Signal Strength (RSS) as a metric for location determination [3]. RSS fingerprints can be easily obtained for most off-the-shelf equipment such as Wireless Fidelity (WiFi) or ZigBee compatible devices.

However, when an electromagnetic wave propagates and encounters objects such as walls and floors, reflections, diffraction, and scattering occur. RSS is influenced not only by the distance but also by any obstacles between the transmitter and receiver [4]. Moreover, it is influenced by multipath effects. Thus, RSS is sensitive to the above factors. When we move on the same floor, the relatively large horizontal errors caused by walls or other obstacles can be tolerated, but too large an error in the height may lead to false floor detection. IPS and their applications are very sensitive to the floor selection [1]. For instance, if a wrong floor is identified, a wrong map is selected to display the position. Determining which floor of the building level one is probably located requires a height measurement with an accuracy of better than 3 m [5].

Traditionally, barometers are used outdoors to measure altitude and meet positioning needs. McLellan [6] presented a method that combined a barometric altimeter and GPS to improve the GPS accuracy and prevent the influence of environment.

To date, micro-electromechanical system (MEMS) pressure sensors can achieve a relatively high accuracy. Massé [7] even used MEMS pressure sensors to study human action (the achieved relative accuracy was 0.53 m). Because of the sensitivity of barometric sensors to vertical movements, a number of researchers [7,8] have used these sensors to detect vertical motions of a human body, such as standing up, sitting down, and falling.

With the miniaturization and decreasing cost of MEMS barometric sensors, they are now installed in a number of portable intelligent devices. MEMS barometric sensors have launched in a number of Android phones such as the Galaxy Nexus, Galaxy SIII, Galaxy Note 2, and other similar devices.

Recent studies on the use of MEMS barometers to increase the accuracy of satellite/inertial navigation system vertical channel are presented in [9,10,11]. These papers showed that MEMS barometers can be successfully integrated with all types of sensors. In [12], a sensor fusion method is presented to track vertical velocity and height based on inertial and barometric altimeter measurements. In [13], smart phones with MEMS barometers were used to detect the difference in altitude in subway stations and commercial centers.

Some studies [1,5,14,15] have presented results on this subject. In [14], data were collected from MEMS barometric sensors under several scenarios where different disturbances affect the pressure readings to determine the relevant error sources of barometers according to the altitude in the field of personal navigation. In [5], the researchers carried out many experiments in this field. The experiments showed the feasibility in detecting the floor level using measurements by mobile device built-in barometric sensors to evaluate the altitude of the mobile devices and then comparing the altitude with the floor heights of the building. These studies were based on the assumption that the accurate floor height in a building is known. However, knowing the heights by direct measurement using instruments or from the architectural blueprints is impossible in some situations. For example, if blueprints are not available and we need to measure the accurate floor heights in a very tall building, measuring it from outside is difficult. Of course, we can measure the distance from the floor to the ceiling in a floor; however, the floor thickness is difficult to measure. To further explore this issue, we conducted an in-depth study on floor positioning without knowing the accurate height of each floor in a building.

The remainder of this paper is organized as follows: in Section 2, we detail the theory and techniques used in the proposed method. In Section 3, we outline the experimental evaluation of the proposed method, and we describe and analyze the results in Section 4. In Section 5, we present our concluding remarks.

## 2. Methods

### 2.1. Theoretical Concepts

Atmospheric pressure has been well known to decrease as the altitude increases. This effect is described by the so-called barometric formula [16]. By using a barometric sensor to measure air-pressure changes, we can calculate the altitude changes corresponding to the pressure. To estimate the altitude of an object relative to a reference point, we utilize the physical phenomenon that barometric pressure decreases with increasing altitude [17]:

(1) $$p=p_{0}\cdot exp\left(-\frac{g\cdot M\cdot h}{R\cdot T_{0}}\right)$$

*p* is the pressure at certain altitude *h*, *p*_0_ is the pressure at a reference point, *M* is the molar mass of dry air, g is the gravitational field strength, *R* is the gas constant of air, and *T*_0_ is the temperature. Air pressure and the amount of water in air change with time, location, and height. From the results of the experiment in [8], temperature compensation has to be considered; however, humidity does not significantly affect the accuracy of the system in indoor altitude estimation. Therefore, we use the gas constant *R* for dry air instead of the gas constant for humid air and the molar mass *M* of dry air instead of the molar mass for humid air. When the altitude changes slightly, we usually consider the gravitational acceleration *g* as constant. *R*, *M* and *g* are given in [17]. Another form is expressed as follows:

(2) $$h=-\frac{R\cdot T_{0}}{g\cdot M}\cdot \ln\left(\frac{p}{p_{0}}\right)$$

### 2.2. Floor Positioning by Barometric Pressure

As mentioned in Section 1, the methods in [1,5,15] are based on the assumption that we know the accurate height of each floor in a building. However, learning the floor heights in some buildings is difficult. Using barometer and temperature measurements as input in Equation (2) will of course allow us to calculate the floor heights; however, the accuracy of this method is not high enough (we studied this problem in a 20-day experiment, and the experimental details are listed in the Appendix). We propose a new approach to determine which floor we are located without knowing the accurate heights of the floors.

**Figure 1:**
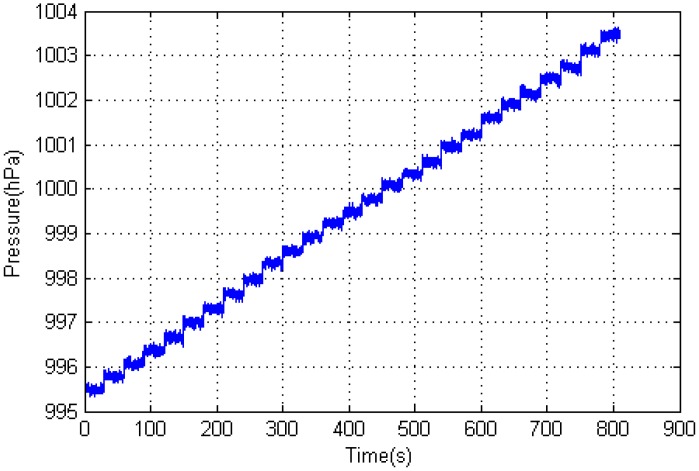
Barometric-pressure distribution of a 25-story building (with two basements). The horizontal axis represents the sampling time (s), and the vertical axis represents the barometric-pressure value (hPa). The data collection order is from the top floor to the basements.

Figure 1 clearly shows a ladder-shaped distribution of the barometric pressure starting with a higher pressure at the lower floor. Using Equation (2), we can calculate the altitude using the pressure values from the built-in barometric sensors of smart phones and the temperature from a local weather station [18]. However, in indoor positioning, we are not interested on the altitude of a certain floor. What we want to know is which floor we are located. If we can learn the thresholds of the pressure ranges from each floor to the next floor upstairs in a building, we can determine which floor we are located by comparing the real-time measurements of the phone built-in barometric sensors with these thresholds.

**Figure 2:**
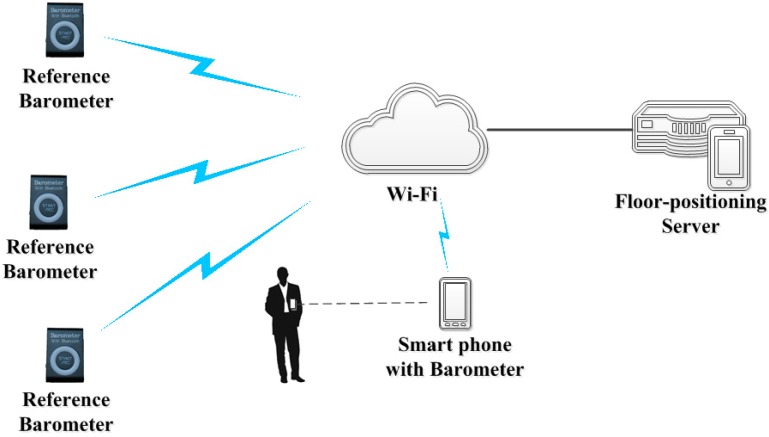
On-line system of floor positioning using multiple reference barometers.

Changes in the atmospheric pressure are generally regular and depend on the season, altitude, and local weather conditions [5]. If real-time pressure thresholds at each floor in a building are required, they can be determined by a direct method of deploying a barometer at each floor. Figure 2 shows that a barometer (called reference barometer) is deployed at each floor to measure the barometric pressure at the floor level (the measurement is called floor pressure threshold *p_thrN_*). When we are at the *N*th floor in a building, the pressure values that we can obtain from the smart phone built-in barometric sensors are always smaller than $p_{thrN}$ and larger than *p_thrN+1_*. We can collect these real-time thresholds via a wireless network and store these thresholds in a floor-positioning server. When we are in a building and carry smart phones, the floor where we are located can be determined by comparing the real-time measurements of the phone built-in barometric sensors with these thresholds obtained from the server through a wireless network. The top and bottom floors have only one threshold (lower and upper thresholds, respectively), and each of the other floors has two thresholds that needs to be compared. We call this floor-positioning method multi-reference barometer floor positioning (MBFP).

Because all comparisons between the pressure values are made without computing the height using Equation (2), the MBFP method is robust and less sensitive to factors such as temperature and humidity. As the thresholds are updated by the reference barometers in time, the changes in the barometric pressure in same floor and the difference in the barometric-pressure change trends at different floors are reflected in these thresholds.

### 2.3. Measurement Process

Because measurements by barometric sensors are unstable, processing these measurements is important. The process includes excluding the outliers and compensating for offsets.

#### 2.3.1. Excluding Outliers

Errors in barometric-pressure estimation may result from factors such as electrical noise, local environmental changes (thermal gradients and sudden air flow [16,19]), and/or platforms of smart phones (software and/or hardware). The methods presented in [19] were able to decrease the influence of noise. However, the problems are the outliers in the measurements. When these outliers are not removed, mutations in the floor-positioning estimation will occur, which will greatly affect the positioning stability. Therefore, judgment and processing questionable data in the measurement sequences are essential in floor positioning. We use the vertical maneuvering ranges of our research objects (smart phone users) as thresholds to eliminate the outliers.

First, we select an appropriate time window, as mentioned in Section 2.3.2. The maximum vertical velocity of the research objects (on stairs or escalators) is *v_max_*. In the current time window (with width *t_w_*), the vertical difference between any measurement point *i* and any point in the previous time window must not exceed ∆*h_max_*:

(3) $$∆h_{max}=2\cdot v_{max}\cdot t_{w}$$

After the current environment temperature *T*_0_ is input, we set *h* = ∆*h_max_*, and using the mean of the processed measurements in the previous time window as *p*_0_, we can calculate the difference in pressure ∆*p_max_* corresponding to ∆*h_max_* using Equation (1). *p_i_* is the measurement in the current window. If |*p_i_*−*p_0_*| > ∆*p_max_*, we consider *p_i_* is an outlier that must be removed; otherwise, it is retained.

In the first time window or when the data collection process stops and restarts, no previous window is considered. Therefore, we need to choose another method for the outliers in these time windows. Barometric pressure varies over time; however, over a short period (time window), it is relatively stable (less than 0.1 hPa for every 10 min [5]). The number of records in a time window is limited. Under these conditions, Grubbs’ test [20,21] is an ideal choice to detect and exclude the outliers. Hence, we use the Grubbs test to eliminate the outliers in the first time window. Certainly, the data cannot be approximated by normal distribution when our research object moves vertically at high-speed (on stairs, escalators, or in elevators) in the first time window. Under this condition, the Grubbs test will remove the motion data from the time window (because the residual of the data is too large), and the data will not consider the floor position. Although some vertical movement details are lost, it is reasonable because the movement between floors is outside our concern. As mentioned in article [22], when we apply Grubbs’ test in our setting, we need to make an extension. Since Grubbs’ test detects one outlier at a time, we can expunge the detected outlier from data of the first time window and iterate the test over the remaining data until no outliers can be found. In this way we can detect multiple outliers.

In this paper, we used $v_{max}$ = 1 m/s. This speed is slightly faster than the speed of escalators (0.3–0.6 m/s [23]) because some people may walk on escalators. Although high-speed elevators likely travel up to 10 m/s [24], we do not consider them because we are not interested on the floors that we pass when we are in elevators.

The current environment temperature $T_{0}$ can be obtained from several sources: local weather station, online weather service [18], temperature sensors of smart phones, and the reference barometer sensors, which will increase the sensitivity of MBFP to temperature, although it is not serious. Using Equation (2), if we set ∆*h_max_* = 2 m and *p_0_* = 1013.25 hPa, the change in temperature from 0 to 15 °C yields the change in *p* from 1013.0098 to 1012.9966 hPa. In other words, the disturbance in the pressure caused by temperature from 0 to 15 °C is 0.0132 hPa, which is less than 0.03 hPa (the noise root mean square (RMS) of BMP180 barometric sensors in ultra-high-resolution mode [25]).

#### 2.3.2. Sampling Frequency and Time Window

The shorter the time window and the response time of the system are, the better is the user experience. The Grubbs test should not be used for sample sizes of six or less [26]. If we want to use a small time window such as 1 s, then the minimum sampling frequency should be 7 Hz to meet the prerequisites of the Grubbs test [20,21]. On the other hand, the fastest sampling frequency of some phone systems or/and barometers is not very high. For example, the sampling frequency of the UG801 smart phone with BMP180 is not higher than 1.5 Hz. In addition, if the sampling frequency is too high, the amount of data collected by the sensors will be too large, creating a burden to the CPU, memory, and battery resources and sharply increasing the data process workload. Accordingly, 10 Hz is a good choose for our sampling frequency. In this article, comparison of the results of the 1-, 3-, and 5-s time windows is presented in the following sections.

#### 2.3.3. Compensation for Offsets

**Figure 3:**
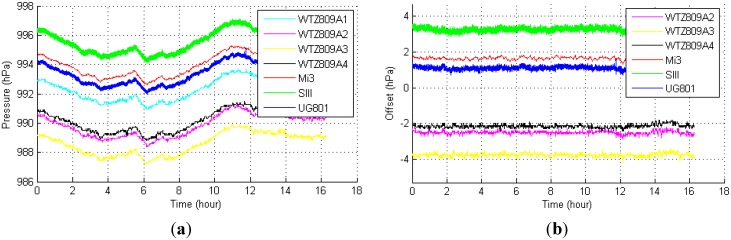
(**a**) Absolute pressure reading from seven sensors. WTZ809A1–WTZ809A4 are barometers with MS5540C, Mi3 and UG901 are smart phones with BMP180, and SIII is a smart phone with LPS331AP; (**b**) WTZ809A1 used as the standard and offsets of the measurements between the other barometers and WTZ809A1.

Different built-in barometric sensors in smart phones may output different measurements although they are at the same altitude and in the same environment. Figure 3 shows the barometric pressure of seven sensors in the same room and at the same altitude. The measurements from different sensors are different. However, in similar environment, the changing trends of pressure values from these sensors are same, in another word, there are almost constant offsets exist among these values [8,14]. Consequently, the offsets have to be compensated by calibration to avoid offset errors. Figure 3b shows the WTZ809A1 used as the standard to calculate the offsets. The detail of these devices is introduced in Section 3.1. The offsets should be measured and compensated regularly to make sure the accuracy of MBFP.

## 3. Experiment

### 3.1. Hardware Selection

To validate our method, a number of commercially available devices were selected.

#### 3.1.1. Smart Phone Selection

The BMP180 (produced by Bosch Sensortec, Kusterdingen, Germany) and the LPS331AP (produced by STMicroelectronics, Mráček) are MEMS pressure sensors widely used in smart phones. To make the experiment more representative, we chose three phones as our test platforms, namely, Mi3, SIII, and UG801, which are equipped with these two sensors (Table 1):

**Table 1:** Test phone details [27,28,29].

	Mi3	SIII	UG801
Barometer	BMP180	LPS331AP	BMP180
OS	Android 4.4.2	Android 4.1.2	Android 4.0.4
Producer	Xiaomi	Samsung	UniStrong

Mi3 and SIII are widely used smartphones. UG801 is an IP65 class ruggedized smart phone for outdoors.

#### 3.1.2. Barometer Selection

We chose a WTZ809A as the reference barometer deployed in the experimental buildings [30]. Table 2 shows the features of WTZ809A.

**Table 2:** WTZ809A features.

Voltage range	DC3.7 V ～ DV5 V
Working current	19 μA ～ 25 μA
Pressure range	10 hPa ～ 1100 hPa
Output	0.1 hPa
Bluetooth	Bluetooth v2.0+
L × W × H	44.08 mm × 29 mm × 13.18 mm

The WTZ809A is a barometer for outdoors sports produced by Waytronic (Shenzhen, China). It uses an MS5540C pressure sensor produced by Measurement Specialties (Hampton, VA, USA). The MS5540C is an SMD-hybrid device, which includes a piezoresistive pressure sensor and an ADC-interface IC. We considered three aspects in selecting the WTZ809A: (1) it can steadily measure barometric pressure and temperature to complete basic experimental tasks; (2) It is very compact (L × W × H = 44.08 mm × 29 mm × 13.18 mm) with built-in battery and is very convenient for deployment (this is particularly important, for example, in a terminal, because big and complex instruments without batteries are difficult to deploy); (3) the data collected by WTZ809A can be sent though Bluetooth or can be stored in the local memory, adapting to different scenarios.

**Figure 4:**
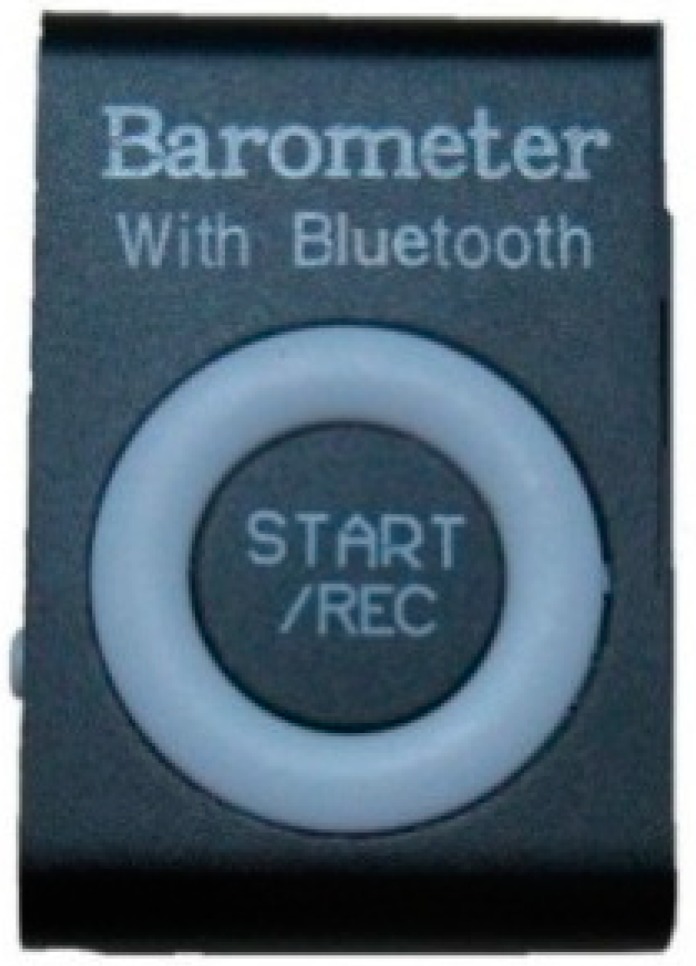
WTZ809A is a portal barometer with Bluetooth. It can work with Android smartphones or PCs though Bluetooth conjunction and can automatically measure, record, and send barometric pressure and temperature at a certain frequency.

The current pressure data collected by four WTZ809A barometers are sent to UG801 phones via Bluetooth. Then, the UG801 phones send the data to a Web Service in a floor-positioning server. If a wireless network is not available for our experiment, such as in airport terminal and other public buildings, built-in memories are used to record the measurements of the WTZ809A barometers (Figure 4). 

To prevent accidental air disturbances, these barometers are placed in holed boxes. Because of the limited battery capacity of the WTZ809A, we have to set the data collection interval to 1 min to ensure that it works for several hours.

### 3.2. Experimental Sites and Procedures

**Figure 5:**
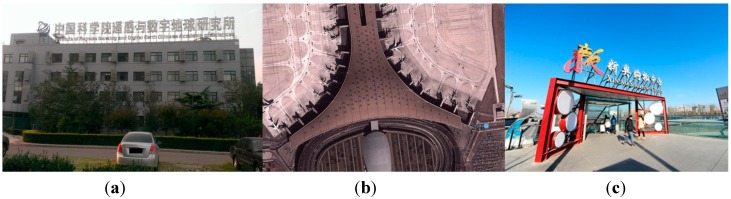
Experimental sites. (**a**) An ordinary office building (**b**) Terminal 3 in Beijing Capital International Airport (from a Google Map screenshot); (**c**) Xin’ao Shopping Center.

We chose three typical buildings as our experimental sites: an office building, an airport terminal, and an underground shopping center (Figure 5).

Mi3 and SIII phones were used in the experiment. The floor numbers were recorded when the subjects were upstairs or downstairs in these buildings to validate our method. In the office building, the Mi3 and SIII phones were placed on an approximately 80-cm-high platform in the hall of each floor during the relatively long experiment. In the terminal and underground shopping center, the subject collected data with the phone in his pocket for half of the experiment time and in his hands for the other half to simulate different conditions. The distance was approximately 80 cm from the ground to the phones in both pockets of the trousers and in the hands. The subject is approximately 168 cm high, and his arms were normally swinging while the phones were in his hands.

#### 3.2.1. Office Building

We chose a four-story office building as one of the experimental sites. It is an ordinary office building in the Olympic Park campus of the Chinese Academy of Sciences. We deployed a WTZ809A barometer at each floor of this building. The barometers recorded the current barometric pressure and temperature once per minute. For each barometer, we used a UG801 phone to collect data via Bluetooth and then transmitted the data to the server through WiFi in the building. The structure is shown in Figure 2. The data were used as barometric-pressure thresholds of the floors.

#### 3.2.2. Terminal

**Figure 6:**
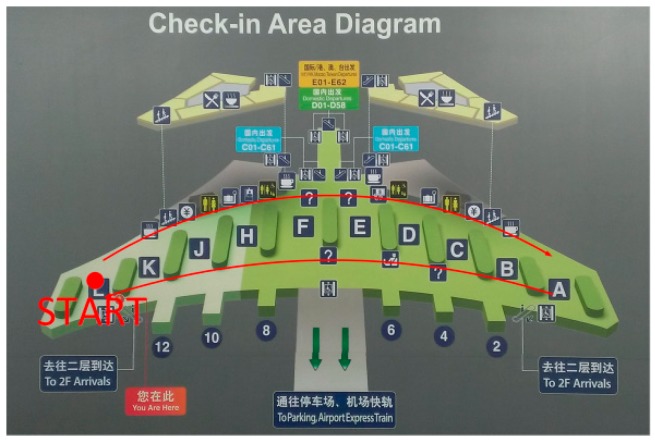
Floor 4 layout of Terminal 3 (the image is from the guide in the terminal). The red lines indicate the route.

Terminal 3 (T3) in the Beijing Capital International Airport (BCIA) is one of the world’s largest integrated terminals [31] where the first, second, and fourth floors and Basement 1 are open for visitors (Figure 6). We selected T3 to test the adaptability of our method in large public buildings on the ground.

T3 has a large hall whose height is higher than general public facilities. To accurately reflect the distribution of the barometer pressure in the hall, the subject walked from one side of the hall to the other side and then back to the starting point. The route intends to cover the entire floor except the security zone.

#### 3.2.3. Underground Shopping Center

Xin’ao Shopping Center is located at an underground court in Beijing Olympic Park. Customers can reach the ground entrance and Basements 1 and 2. We chose this building because it is a large underground facility, which leads to the subway, and is very representative. We can test the adaptability of our method in underground environment.

## 4. Results and Discussion

In this section, we introduce the results of the floor positioning and analyze the factors that affect the results.

### 4.1. Results of the Measurement Process

#### 4.1.1. Outliers and Noise

In [19], the barometric altimeter noise is decomposed into three components with different physical origins, namely global environment changes, local environment changes, and an uncorrelated random process, mainly due to wideband electronic noise, including quantization noise. The global environment changes can be ignored in our experiment because in respectively small time windows, the changes are small [5]. Furthermore, we have to consider the factors from the platforms of smartphones (software and/or hardware).

The results of excluding the outliers in the measurements from the Mi3 with BMP180 and SIII with LPS331AP smart phones were analyzed. We believe that the major outlier sources in the floor positioning included the following factors:

*Type 1*: Outliers caused by the smart phone platforms. This type of outliers often appears on Mi3 phones (probably because the operating system has been redesigned based on Android or their data process algorithms are not perfect). The difference between ordinary measurements and the outliers was very large, which was even more than 100 hPa. It is a major factor that affects the positioning results.

*Type 2*: Outliers generated by environmental factors including temperature, active motion, and/or airflow.

*Type 3*: Outliers due to the sensors themselves (electrical noise) and/or other unknown factors. Even if we placed the phones in a room with calm air and small temperature change, outliers still occasionally appeared. We believe that this type of outliers could be due to the sensors themselves or other unknown factors. The measurement by the SIII with LPS331AP smart phone had a relatively higher probability of occurrence of this outlier type.

In summary, we believe that in the floor-positioning process, Type 1 outliers must be removed because their effect on the positioning results is relatively large. Types 2 and 3 outliers should be excluded if they exceed the thresholds mentioned in Section 2.3.1; otherwise, the data may be retained if they do not exceed the thresholds because they have no serious influence on the positioning results.

**Figure 7:**
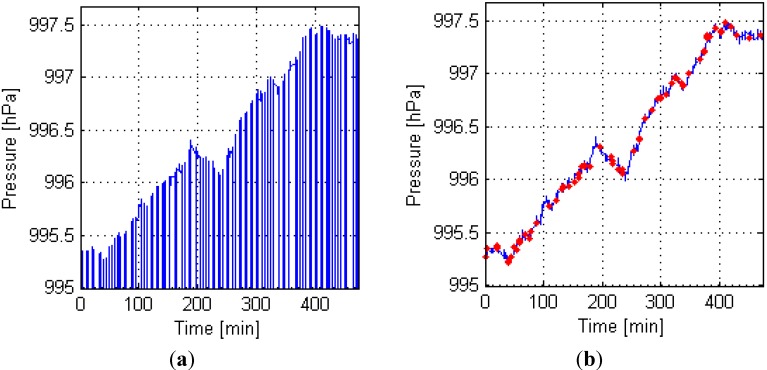
Measurement process by Mi3 with BMP180. (**a**) Raw data; (**b**) Results when outliers are excluded.

In addition, noise is an important factor that influences the positioning results. We use the mean of the measurements (recommended by Bosch Sensortec [25]) in a time window without outliers as an input for the floor positioning to counteract the influence of RMS noise. The following are the details of the test:

Figure 7 shows that the data collection time is approximately 480 min. Figure 7a shows that the Mi3 phone produces many Type 1 outliers (jump points), which may seriously affect the positioning results. The residuals of Types 2 and 3 outliers are relatively large, but they are much lesser than those of Type 1. Therefore, we evaluated the effect of the outlier exclusion mainly based on Type 1. Figure 7b shows that all 80 Type 1 outliers are properly removed; 83 Types 2 and 3 outliers were excluded.

**Figure 8:**
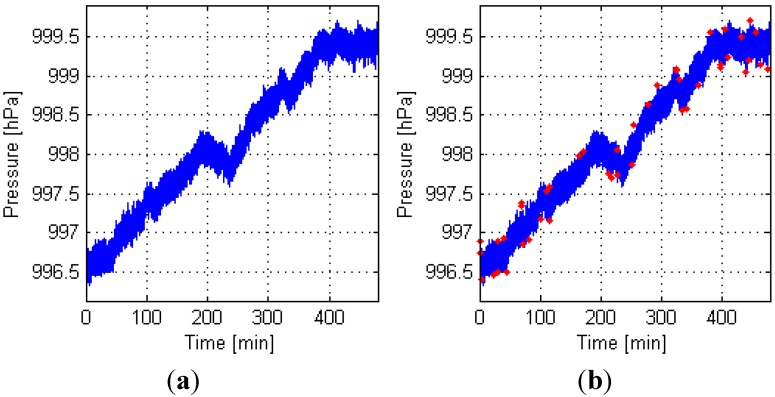
Treatment of the measurements from SIII with LPS331AP. (**a**) Raw data; (**b**) Results when outliers were excluded.

Figure 8 shows that the data collection time is approximately 480 min. Figure 8a shows that the noise of measurements from SIII with LPS331AP is terrible, which is a major factor affecting the floor positioning accuracy (mentioned in Section 4.1). Figure 8 shows that Type 1 outliers did not appear, and 44 Types 2 and 3 outliers were excluded.

#### 4.1.2. Compensation for the Offsets

Figure 3 shows that the measurements of the barometers/sensors of the phones used in our experiment are not the same although they were collected in the same room and at the same altitude. The collection time lasted for approximately 16 h. We used the WTZ809A1 and UG801 measurements as standards to calculate the offsets. The means and standard deviations of the offsets are listed in Table 2 and Table 3.

**Table 3:** Means and standard deviations of the offsets using WTZ809A1 as standard.

**Barometer**	**WTZ809A2**	**WTZ809A3**	**WTZ809A4**
Mean (hPa)	−2.4997	−3.7518	−2.1368
Std.	0.0990	0.1015	0.1029
**Phone**	**Mi3**	**SIII**	**UG801**
Mean (hPa)	1.6386	3.2461	1.0925
Std.	0.0890	0.1126	0.0899

**Table 4:** Means and standard deviations of the offsets using UG801 as standard.

**Barometer**	**WTZ809A1**	**WTZ809A2**	**WTZ809A3**	**WTZ809A4**
Mean (hPa)	−1.0937	−3.5946	−4.8467	−3.2321
Std.	0.0943	0.0873	0.1038	0.1117
**Phone**	**Mi3**		**SIII**	
Mean (hPa)	0.5490		2.1535	
Std.	0.0501		0.0828	

As mentioned in Section 2.3.1, the offsets among the pressure measurements are nearly constant. When these barometers were deployed on the floors in the experimental sites, we used the means of these offsets to compensate for the measurements by the barometers. The standard deviations of the offsets are related not only to the standard barometer but also to the other phone barometers/sensors.

#### 4.1.3. Comparison between Different Standards 

The means and standard deviations listed in Table 3 considered a WTZ809A with MS5540C as a standard. The corresponding standard in Table 4 is a UG801 with BMP180. Most standard deviations in Table 4 are less than the corresponding values listed in Table 3. Although we chose WTZ809A as the standard barometer because it is easily deployed in public environments, the UG801 with BMP180 yielded higher precision. An offset of 0.1 hPa, for example, causes an error of approximately 0.84 m at normal temperature according to Equation (2). Because the story height of most buildings is approximately 3 m [32], either UG801 or WTZ809A is acceptable as the standard barometer.

#### 4.1.4. Comparison between Different Barometers/Sensors of Phones 

Table 3 shows that the difference in the standard deviations of Mi3 and UG801 is small because they used the same sensor BMP180. The standard deviation of SIII with LPS331AP is larger than the corresponding values of Mi3 and UG801. We hypothesized that phones with BMP180 would achieve higher accuracy than those with LPS331AP in floor positioning, and the result presented in Section 4.2.1 confirms our hypothesis.

### 4.2. Positioning Results and Analysis

In this section, we present the comparison and analysis of the results from different time windows, phone types, phone-carrying modes, and experimental sites to understand the effect of these factors on the floor positioning. To perform more accurate analysis of the experimental results, data from upstairs and downstairs were excluded.

#### 4.2.1. Different Time Windows and Phone Types

We used 1, 3, and 5 s as time windows to determine the floor position in the three sites. Both Mi3 and SIII were used in the experiment. The results are listed in Table 5.

**Table 5:** Results of the floor positioning with different time windows and phones.

Time Window	1 s (%)	3 s (%)	5 s (%)
Both	95.65	95.99	96.06
Mi3	98.53	98.58	98.62
SIII	92.10	92.79	92.89

The results listed in Table 5 show that using the mean of a larger time window can better eliminate the RMS effect, and the accuracy can be slightly improved; however, the downside is a slower response time of the system and consumption of a little more random access memory of phones. Certainly, larger time windows lead to poor user experience. Overall, no large difference was observed in the positioning accuracy under different time windows. As mentioned in Section 4.1, the RMS of Mi3 is smaller than that of SIII. The results listed in Table 5 also demonstrate that phones with smaller RMS yield better positioning results.

#### 4.2.2. Different Phone-Carrying Modes

We used 1, 3, and 5 s as time window for floor positioning in two sites: the terminal and underground shopping center. Two phone-carrying modes were considered: in the trouser pockets and in the hands. Because the office building was the site for a long-time fixed experiment, the data collected there were excluded in this section. Both Mi3 and SIII were used in the experiment. Table 6 lists the results.

**Table 6:** Floor-positioning results under different carrying modes and time windows.

Time Window	1 s (%)	3 s (%)	5 s (%)
In the pockets	95.50	95.53	95.48
In hands	99.42	99.48	99.56

Surprisingly, the accuracy of the data collected from the phones in the hands is higher than that in the pockets. When the phones are in the subject hands, the barometric sensors are affected by the arm activities and airflow. When the phones are in the pockets, the influence of outside airflow is reduced. However, other factors may have affected the measurements such as pressure caused by relative motions and/or different temperature in the pockets.

#### 4.2.3. Different Experimental Sites

In this section, we present the comparison of the positioning results in the different experimental sites. In the office building, a long-time fixed experiment was performed, whereas in the terminal and underground shopping center, the experiments were performed by a subject using smart phones. The results are listed in Table 7.

**Table 7:** Floor-positioning results under different carrying modes and time windows.

Sites	Phones	1 s (%)	3 s (%)	5 s (%)
Office building	Mi3	98.54	98.58	98.62
	SIII	92.10	92.79	92.89
Terminal	Mi3	92.63	92.57	92.65
	SIII	88.76	88.74	88.31
Underground shopping center	Mi3	96.23	96.35	96.09
	SIII	98.06	98.03	98.29

#### 4.2.4. Office Building

**Figure 9:**
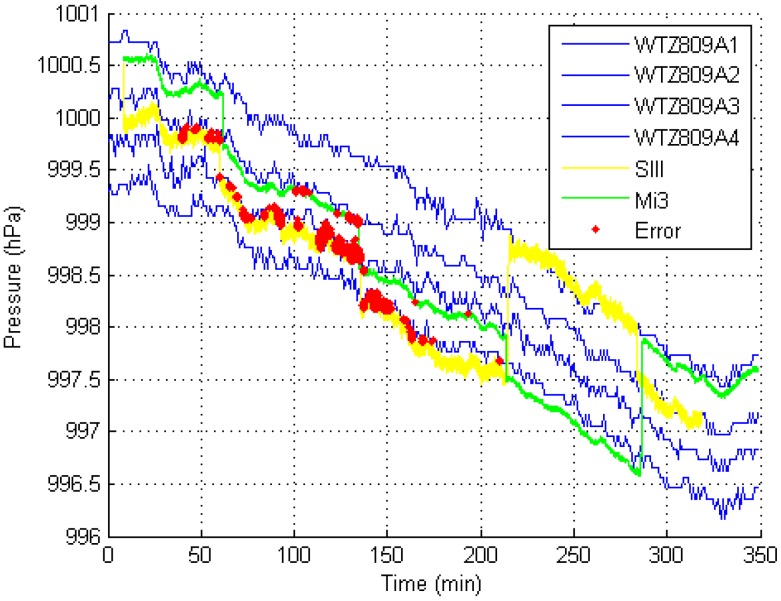
Experimental result in the office building (using a 1-s time window).

The experiment in the office building took approximately 6 h to execute. Figure 9 shows the experimental result.

In the experiment, the phones were placed at the floor for 60–90 min and then moved to other stories. Because the phones were placed on a platform in the hall at each floor, the influence of the arm and leg motions and the environment in the pockets were excluded. The experimental results indicate that our method met the basic need for floor positioning in a building for a relatively long time.

#### 4.2.5. Terminal

**Figure 10:**
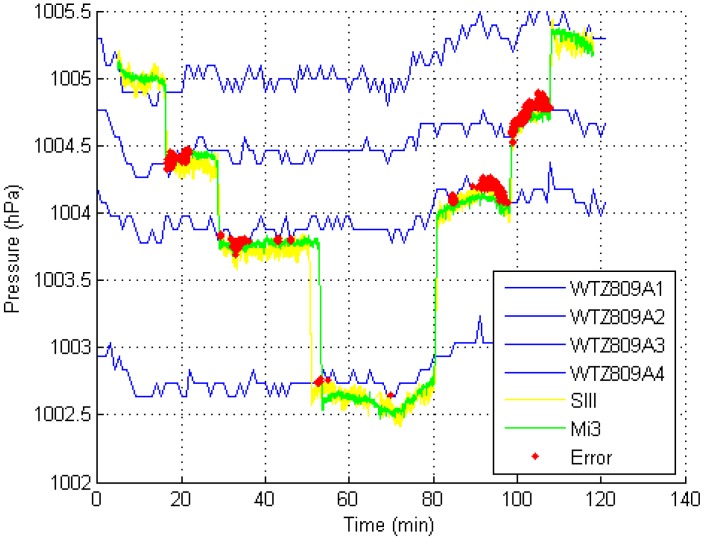
Experimental result in the terminal (using a 1-s time window).

The experiment in the terminal took approximately 2 h to perform. Figure 10 shows the experimental result.

In the experiment, an adult male subject placed Mi3 and SIII phones in different pockets in his trousers and started from Floor B1 along the route shown in Figure 6 to collect data. After completing the data collection from Floors B1 to 4, the phones were removed from his pockets, and the subject continued to collect data from Floors 4 to B1. The paths of Floor 2 and 4 are longer than these of Floors 1 and B1. At Floors 2 and 4, the subject took 15–20 min to cover the routes. From the experimental results, although the terminal contains a large indoor area, the method achieved satisfactory positioning accuracy.

#### 4.2.6. Underground Shopping Center

**Figure 11:**
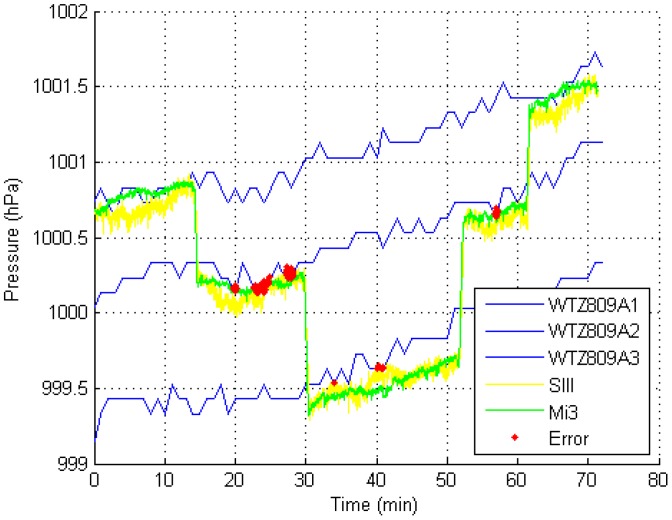
Experimental result in the underground shopping center (using a 1-s time window).

The experiment in the underground shopping center took approximately 1 h to perform. Figure 11 shows the experimental result.

The data collection process is similar to that in the terminal. The experimental results show that our method can adapt to a large underground indoor-area environment with air conditioners.

#### 4.2.7. Analysis of the Results

*Necessity of using multiple reference barometers.* The blue lines in Figure 9, Figure 10 and Figure 11 represent the barometric-pressure change trends from four reference barometers. The trends are similar but are not the same. Because a difference exists between the change trends in the barometric pressure at each floor, deploying a reference barometer at each floor is necessary to accurately observe the changes in pressure at each floor, which yields more accurate thresholds. The methods in [1,4,15] did not consider the difference in the barometric-pressure change trends at different floors. Compared with these conventional methods, the MBFP method requires more barometers, which is logical. At present, the use of many barometers is not a big problem. MEMS barometers are considerably inexpensive. For example, the WTZ809A used in our test costs no more than USD $30.00. Because we cannot determine the accurate floor heights in the experimental sites, comparing the results of those methods with those of our method is quite impractical.

*Influence of the reference barometer working mode.* Because of limited battery capacity, we have to set the data collection interval of the reference barometers to 1 min (mentioned in Section 3.1.2), which affects the positioning accuracy of the MBFP. Figure 9, Figure 10 and Figure 11 show that the curves of the reference barometers are not smooth. In practice, external power should be supplied to the reference barometers to increase the data collection frequency.

*Selection of the time window*. The characteristic of the different time-window sizes has already been mentioned in Section 4.2.1. We tend to use a smaller time window because a smaller time window leads to better user experience. In contrast, large time window cannot conspicuously improve the positioning accuracy.

*Influence of air conditioners.* In our experiment, the office building did not have a central air conditioner, and the windows in the halls at each floor were open. The terminal and the underground shopping center were equipped with air conditioners. From the experimental results, we did not find decisive influence of the air conditioners on the floor positioning.

*Influence of the site area.* Compared with the other two relatively small experimental sites, the positioning accuracy in the terminal was lower. A possible reason could be that we only deployed a barometer at a floor with a very large area, and one barometer is not sufficient to detect the precise distribution of barometric pressure. This problem can be solved by deploying more barometers in one floor. Of course this is related to the plane indoor positioning, which is not the focus of this paper.

## 5. Conclusions

In this paper, we proposed a method (MBFP) of using multiple reference barometers for floor positioning in buildings. Compared with the previous methods that are completely based on the barometric formula, the MBFP method has the following characteristics:

- It does not need to know the accurate heights of the buildings and stories; therefore, it can be more widely applied.
- It is robust and less sensitive to temperature, humidity, and other similar factors.
- It considers the difference in the barometric-pressure change trends at different floors and is more reasonable.

We selected three typical experimental sites, namely, an office building, an airport terminal, and an underground shopping center, as sites of our experiments. These sites include ground-level and underground facilities, large public buildings, typical office environments, and architecture with or without air conditioners. In the experiment, the phones were placed on platforms and/or carried in pockets and hands to simulate different carrying and utility modes. The experimental results show that our MBFP method performed well under these conditions. The required experimental equipment is common, inexpensive, and ubiquitously used, which validates the good adaptability of our method. In our future work, we intend to focus on the combination of MBFP with other methods that can provide indoor plane location to improve the indoor positioning precision.

## Appendix

Is it valid to measure the accurate heights of the floors of buildings using Equation (2)? To find the answer to this question, we have consecutively collected barometric-pressure distribution data of a 25-floor building (with two basements) for 20 days. We used SIII with LPS331AP and Mi3 with BMP180 smart phones in our observation. The observation was conducted from 22 June 2014 to 22 July 2014. To reduce the effect of human activities on the measurements, the data were collected daily at 23:00. Data from the top floor to the basement were collected. The phones were placed on platforms 1 m high from the ground at each floor. The data collection time at each floor lasted for 30 s, and the sampling frequency was 10 Hz. The daily observation took approximately 40 min. The temperature data required by Equation (2) were obtained from t7online [18]. The actual floor heights of the building were obtained from the blueprint of the building.

Because the 25th floor is the top floor and we cannot reach the roof, we can only calculate the heights of the 1st–24th floor and Basements 1 and 2. The top floor height is of course unimportant in floor positioning. Because the duration of the daily collection was approximately 40 min and the sea level barometric pressure was volatile at this time, we used the means of the measurements (the outliers had been removed) at two adjacent floors as the input for Equation (2) to calculate the floor heights. The interval between two adjacent measurements was approximately 1 min, and the environment barometric pressure only slightly changed.

Table A1 reveals that the accuracy and precision of the measurement are largely related to the sensors. The accuracy and precision from the SIII with LPS331AP measurements are lower than those from the Mi3 with BMP180. The maximum standard deviation using LPS331AP reached 0.446, which shows a bad precision. Table A1 shows that the measured floor heights are the mean of the 20-day observation. They were the results of iterative observations, which enhanced the accuracy. In our log, the maximum difference between the measured and actual heights are very large (LPS331AP, 1.631 m; BMP180, 1.041 m) in a one-day observation. Obviously, for a large number of buildings, performing a 20-day observation to reduce errors is difficult.

In addition, the differences between the heights from the observations and blueprint vary according to floors. The differences do not show apparent trends. We believe that they might be caused by different local environments. This difference demonstrates the necessity of using multiple reference barometers mentioned in Section 4.2.4. In summary, we believe that when the barometric pressure from the built-in pressure sensors of typical smart phones are used as input of Equation (2), we can roughly estimate the floor height of buildings. These rough floor-height estimates may lead to large floor-positioning errors.

**Table A1:** Floor heights of the building. Height BMP180 is the 20-day mean of the measured height by BMP180, and Std. BMP180 indicates the standard deviation of Height BMP180. Height LPS331AP is the 20-day mean of the measured height by LPS331AP, and Std. LPS331AP is the standard deviation of Height LPS331AP. Design Height is the actual floor height obtained from the blueprint.

Floor	Height (m) BMP180	Std. BMP180	Height (m) LPS331AP	Std. LPS331AP	Design Height (m)
B2	3.556	0.288	3.580	0.312	3.500
B1	3.220	0.240	3.193	0.306	3.200
1st	2.709	0.211	2.653	0.353	2.700
2nd	2.585	0.236	2.688	0.373	2.700
3rd	2.521	0.221	2.556	0.446	2.700
4th	2.720	0.151	2.687	0.243	2.700
5th	2.880	0.327	2.929	0.353	3.000
6th	2.726	0.229	2.665	0.257	2.700
7th	2.655	0.201	2.671	0.380	2.700
8th	2.613	0.185	2.497	0.278	2.700
9th	2.610	0.294	2.700	0.341	2.700
10th	2.619	0.198	2.606	0.275	2.700
11th	2.616	0.213	2.618	0.245	2.700
12th	2.642	0.230	2.605	0.360	2.700
13th	2.664	0.208	2.673	0.339	2.700
14th	2.681	0.197	2.651	0.393	2.700
15th	2.654	0.130	2.662	0.278	2.700
16th	3.113	0.150	3.151	0.329	3.100
17th	2.642	0.143	2.637	0.240	2.700
18th	2.685	0.164	2.624	0.293	2.700
19th	2.665	0.133	2.624	0.311	2.700
20th	2.571	0.222	2.455	0.420	2.700
21st	2.633	0.181	2.795	0.316	2.700
22nd	2.566	0.228	2.674	0.350	2.700
23rd	2.616	0.210	2.548	0.397	2.700
24th	2.758	0.184	2.581	0.429	2.700

## References

[1] Wang H., Lenz H., Szabo A., Hanebeck U.D., Bamberger J. Fusion of barometric sensors, wlan signals and building information for 3-d indoor/campus localization. Proceedings of the International Conference on Multisensor Fusion and Integration for Intelligent Systems (MFI 2006).

[2] Galvan-Tejada C.E., Garcia-Vazquez J.P., Brena R.F. (2014). Magnetic field feature extraction and selection for indoor location estimation. Sensors.

[3] Wu C., Yang Z., Liu Y., Xi W. (2013). Will: Wireless indoor localization without site survey. IEEE Trans. Parallel Distrib. Syst..

[4] Xi S., Kun X., Xiaoqiang S., Jian W., Jintong L. Optimized indoor wireless propagation model in wifi-rof network architecture for rss-based localization in the internet of things. Proceedigns of the Microwave Photonics, 2011 International Topical Meeting on & Microwave Photonics Conference.

[5] Binghao L., Harvey B., Gallagher T. Using barometers to determine the height for indoor positioning. Proceedings of the 2013 International Conference on Indoor Positioning and Indoor Navigation (IPIN).

[6] McLellan J.F., Schleppe J., McLintock D., Deren G. Gps/barometry height-aided positioning system. Proceedings of the 1994 IEEE Position Location and Navigation Symposium.

[7] Massé F., Bourke A.K., Chardonnens J., Paraschiv-Ionescu A., Aminian K. (2014). Suitability of commercial barometric pressure sensors to distinguish sitting and standing activities for wearable monitoring. Med. Eng. Phys..

[8] Bollmeyer C., Esemann T., Gehring H., Hellbruck H. Precise indoor altitude estimation based on differential barometric sensing for wireless medical applications. Proceedings of the 2013 IEEE International Conference on Body Sensor Networks (BSN).

[9] Retscher G. Location determination in indoor environments for pedestrian navigation. Proceedings of the 2006 IEEE/ION Position, Location, And Navigation Symposium.

[10] Seo J., Lee J.G., Park C.G. Bias suppression of gps measurement in inertial navigation system vertical channel. Proceedings of the PLANS 2004 Position Location and Navigation Symposium.

[11] Landis D., Thorvaldsen T., Fink B., Sherman P., Holmes S. A deep integration estimator for urban ground navigation. Proceedings of the PLANS 2006, IEEE/ION Position, Location and Navigation Symposium.

[12] Sabatini A., Genovese V. (2014). A sensor fusion method for tracking vertical velocity and height based on inertial and barometric altimeter measurements. Sensors.

[13] Zegarra Flores J., Farcy R., Miesenberger K., Fels D., Archambault D., Peňáz P., Zagler W. (2014). Indoor navigation system for the visually impaired using one inertial measurement unit (IMU) and barometer to guide in the subway stations and commercial centers. Computers Helping People with Special Needs.

[14] Parviainen J., Kantola J., Collin J. Differential barometry in personal navigation. Proceedings of the 2008 IEEE/ION Position, Location and Navigation Symposium.

[15] Bai Y., Jia W., Zhang H., Mao Z.H., Sun M. Helping the blind to find the floor of destination in multistory buildings using a barometer. Proceedings of the 2013 35th Annual International Conference of the IEEE, Engineering in Medicine and Biology Society (EMBC).

[16] Berberan-Santos M.N., Bodunov E.N., Pogliani L. (1997). On the barometric formula. Am. J. Phys..

[17] NASA N., Force U.A. (1976). Standard Atmosphere 1976.

[18] T7online. http://www.t7online.com.

[19] Sabatini A., Genovese V. (2013). A stochastic approach to noise modeling for barometric altimeters. Sensors.

[20] Grubbs F.E. (1969). Procedures for detecting outlying observations in samples. Technometrics.

[21] Grubbs F.E. (1950). Sample criteria for testing outlying observations. Ann. Mathemat. Stat..

[22] Yang Y., Wang X., Zhu S., Cao G. (2008). Sdap: A secure hop-by-hop data aggregation protocol for sensor networks. ACM Trans. Inf. Syst. Secur..

[23] Wikipedia Escalator. http://en.wikipedia.org/wiki/Escalator.

[24] Wikipedia Elevator. http://en.wikipedia.org/wiki/Elevator.

[25] Bmp180 Datasheet. http://ae-bst.resource.bosch.com/media/products/dokumente/bmp180/BST-BMP180-DS000-09.pdf.

[26] Garcia F.A.A. (2012). Tests to Identify Outliers in Data Series. Ph.D. Thesis.

[27] Mi 3. http://www.mi.com/en/mi3.

[28] Siii. http://www.samsung.com/hk_en/consumer/mobile/mobile-phones/smartphone/GT-I9300RWITGY.

[29] UG801. http://www.unistrong.com/Member/ProShow.aspx?proid=UG801.

[30] Multifunctional barometer. http://www.wt-smart.com/en/show.asp?id=40.

[31] BCIA Introduction. http://en.bcia.com.cn/aboutus/index.shtml.

[32] Wikipedia Storey. http://en.wikipedia.org/wiki/Storey.

